# Supplementary material for: New avenues for potentially seeking microbial responses to climate change beneath Antarctic ice shelves
Source: mSphere. 2024 Apr 26;9(5):e00073-24. doi: 10.1128/msphere.00073-24 (PMC11237435; doi:10.1128/msphere.00073-24)
Supplement: Supplemental material — Supplemental figures and tables. [file msphere.00073-24-s0001.docx]

Supplementary Material for

New avenues for potentially seeking microbial responses to climate change beneath Antarctic ice shelves

Aitana Llorenç Vicedo^1,2^, Monica Lluesma Gomez^1,2^, Ole Zeising^3^, Thomas

Kleiner^3^, Johannes Freitag^3^, Francisco J. Martínez-Hernández^1^, Frank

Wilhelms^3^, and Manuel Martínez-García^*1,2^

^1^Department of Physiology, Genetics, and Microbiology, University of Alicante, Carretera San Vicente del Raspeig, San Vicente del Raspeig, Alicante, 03690, Spain

^2^Multidisciplinary Instititue for Environmental Studies (IMEM), University of Alicante, Carretera San Vicente del Raspeig, San Vicente del Raspeig, Alicante, 03690, Spain

^3^Alfred-Wegener-Institut Helmholtz-Zentrum für Polar-und Meeresforschung,

Bremerhaven, Germany

This supplementary material contains:

-Supplementary Figures 1-8

-Supplementary Table 1-2

-Supplementary References

# Supplementary Figures


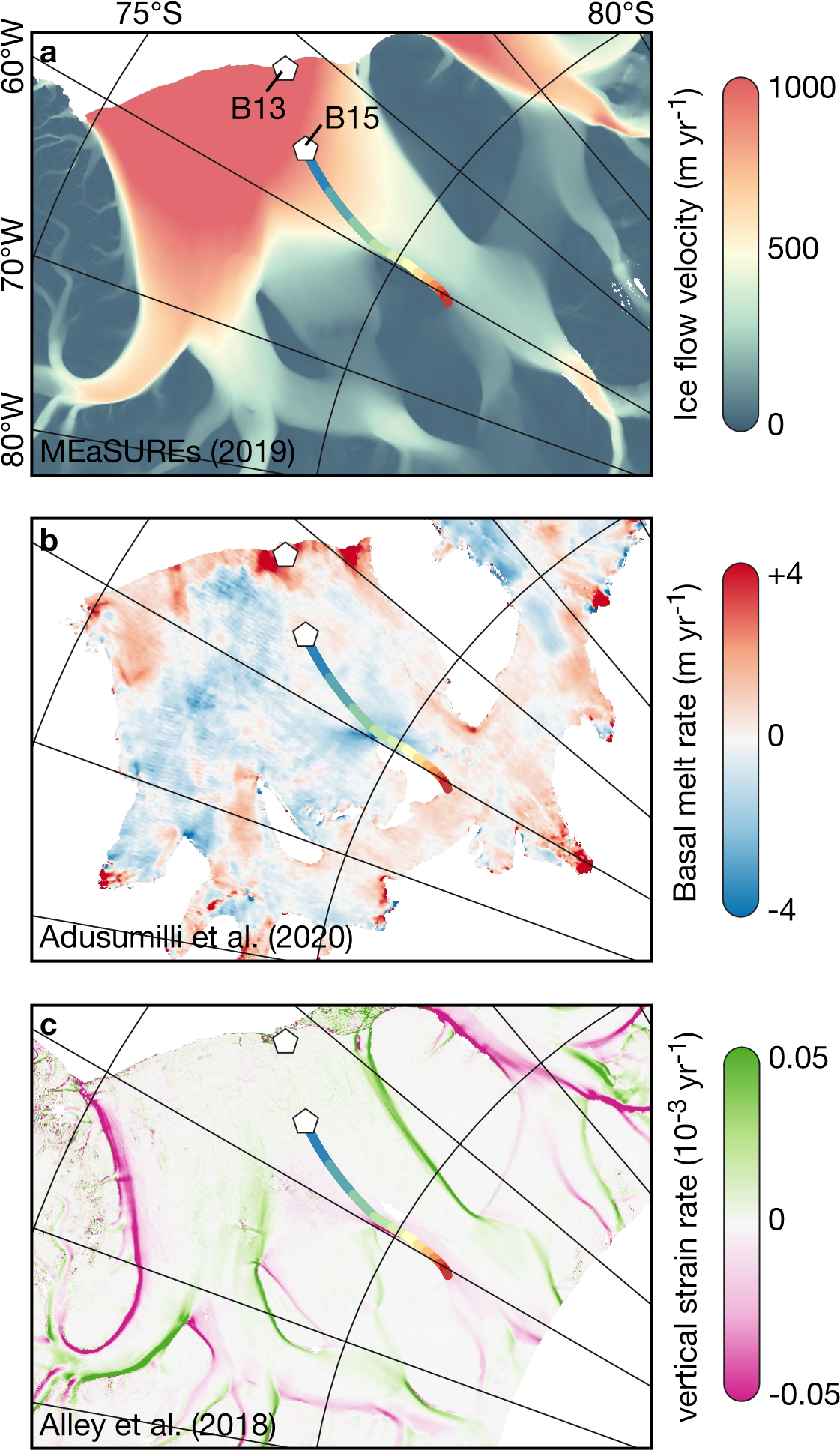
 drilling (yr)

0 - 100

100 - 200

200 - 300

300 - 400

400 - 500

500 - 600

600 - 700

700 - 800

800 - 900

900 - 1000

1000 - 1100

1100 - 1200

**Figure S1**: Observational data sets used for age-depth relation of marine ice at B15. **a** Map of ice flow velocities of Ronne Ice Shelf from MEaSUREs ^1,2^ with flowline of B15. The color of the flowline shows the flow duration before drilling. **b** Map of basal melt rates from Adusumilli et al.^3^ **c** Map of vertical strain rates from Alley et al.^4^


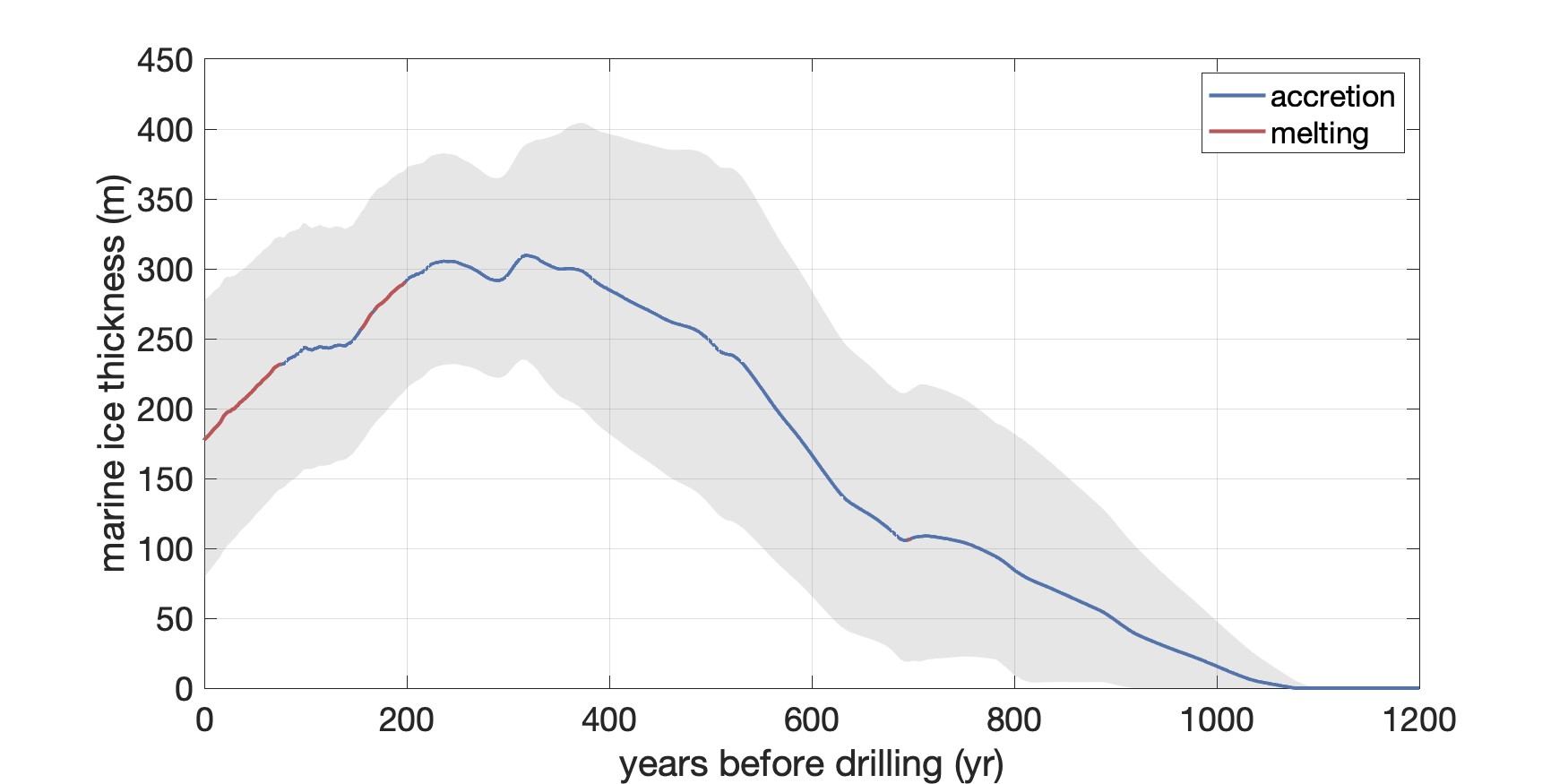


**Figure S2**: Evolution of marine ice thickness along the flowline of B15. The color shows time periods of accretion and melting, the grey shaded area shows the uncertainty.


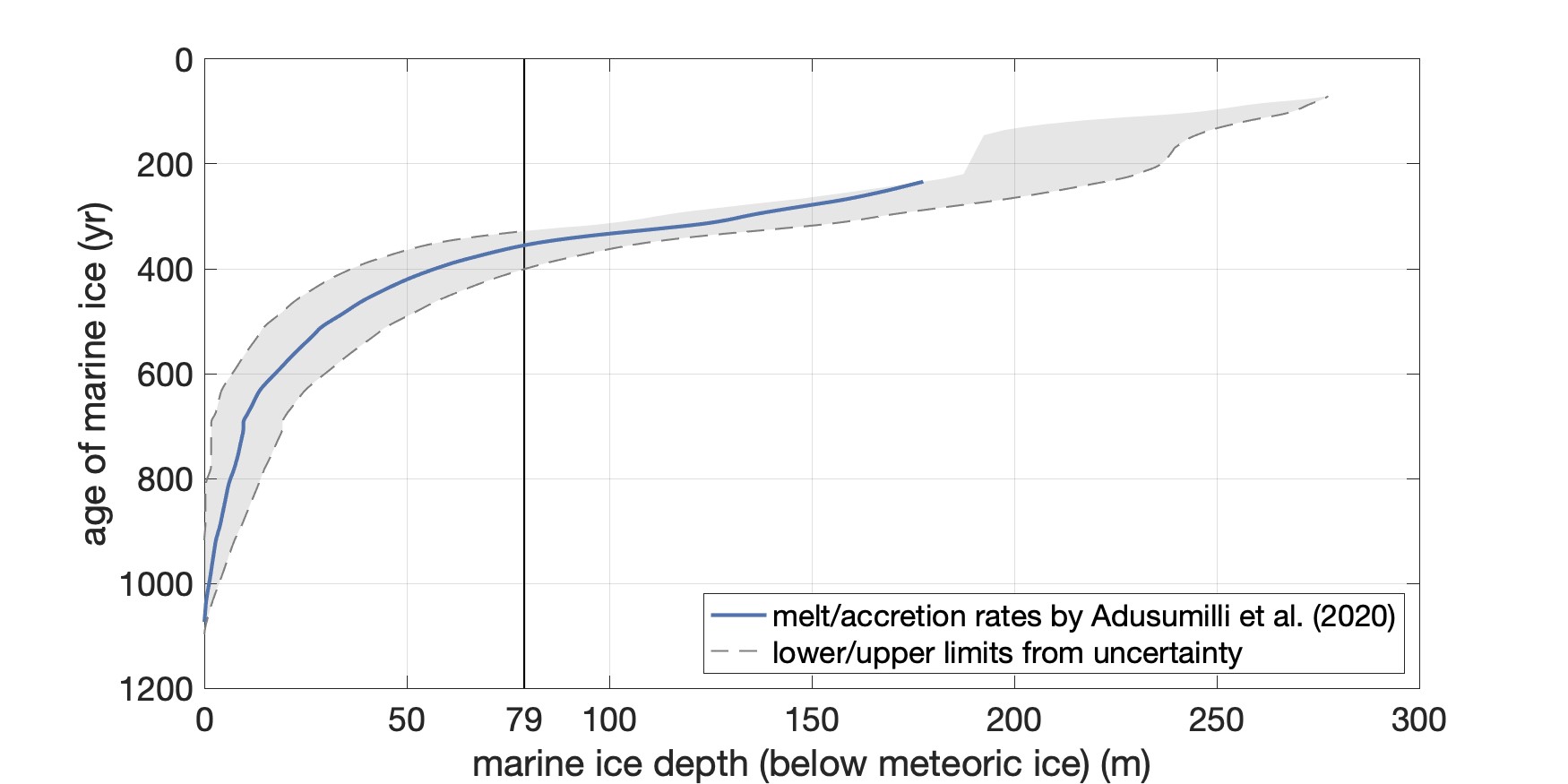


**Figure S3**: Age-depth relation of marine ice aggregated at B15 location. The grey shaded area displays the range between the upper and lower limits of the cumulated uncertainty of age caused by uncertainties in the estimate of basal melt/accretion rates along the modelled flow line. Note that melt/aggregation rates are assumed as constant over time.


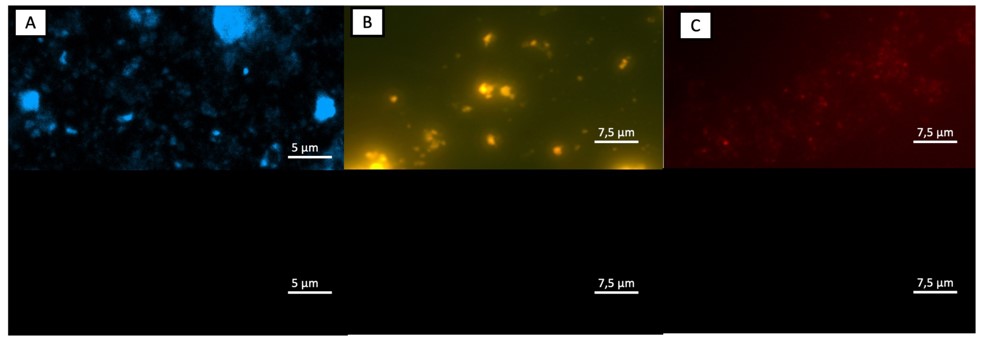


**Figure S4**. Images in bottom showing no autofluorescence of unstained marine ice samples in the blue, green-yellow and red fluorescence channel. Unstained samples of marine ice core were visualized under confocal microscopy Leica SP2 with identical conditions of laser power and gain than that used for stained samples. Data from unstained samples indicating that these particles themselves did not show autofluorescence, albeit, as described before (see refs. In main text), DNA fluorescent dye interacts unspecifically with organic and inorganic particles, which. Might explain many of the amorphous observed structures showing fluorescence (top images).


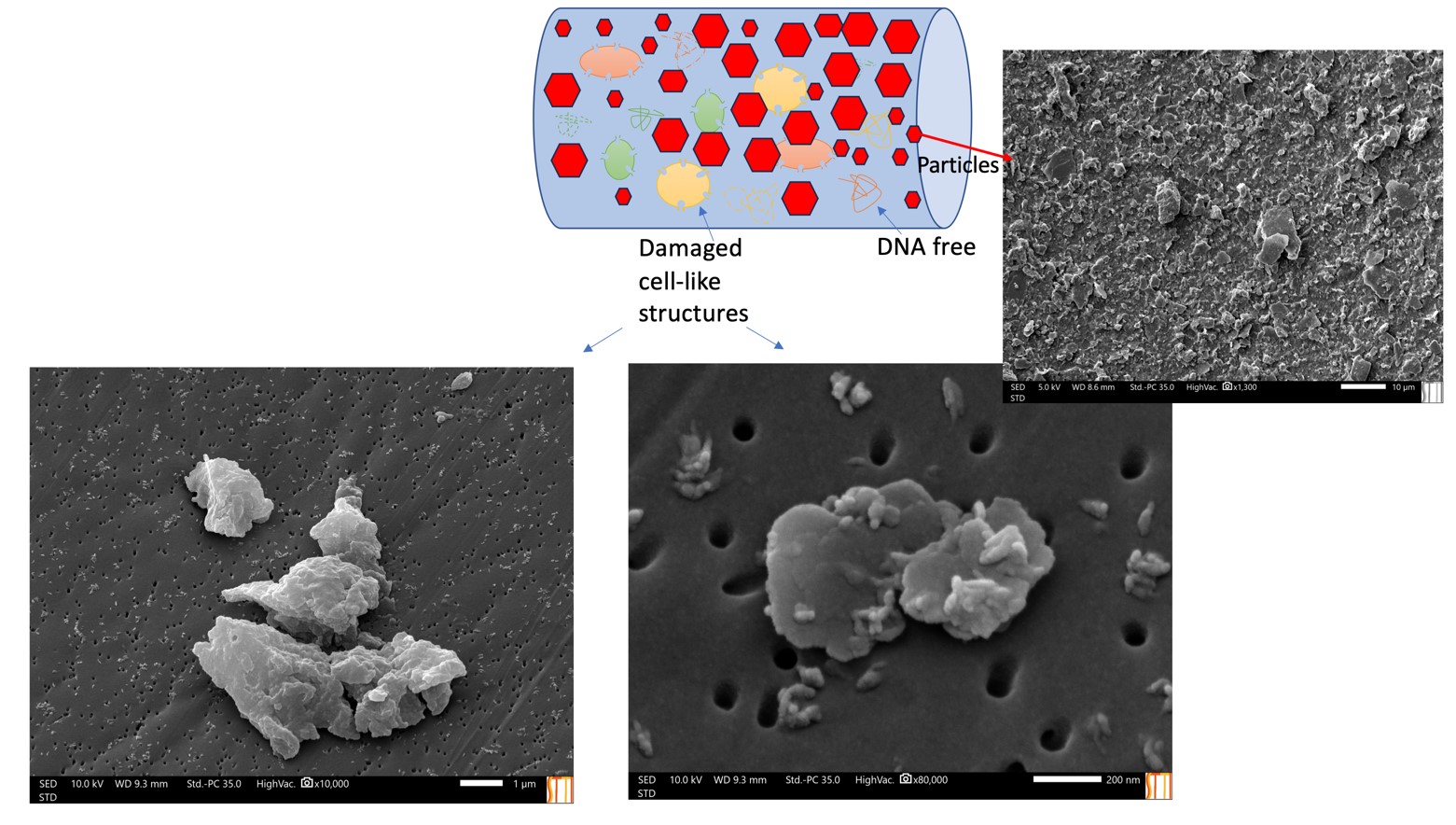


**Figure S5.** Scanning electron images of the marine ice core B15. Most of the observed structures were likely particle (top picture), In addition, some lysed/damaged cell-like structures like the one shown in the bottom images were also observed at very low concentration. SEM data supports that most of the stained fluorescent structures observed with fluorochrome dyes by confocal microscopy were indeed particles and likely most of the cells preserved after 400 years old were already damaged and likely lysed during melting of ice core and processing for microscopy.


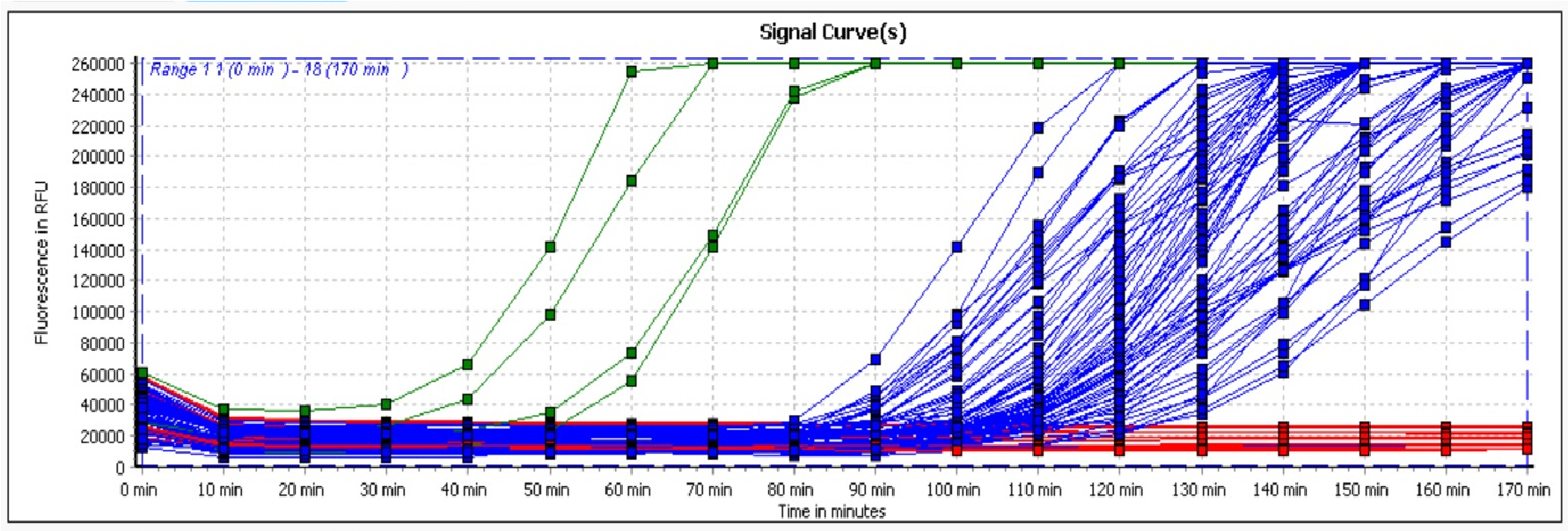


**Figure S6.** Real time multiple displacement amplification of inner ice core sample. Well containing melted ice core are shown in blue color. Negative controls of MDA are displayed in red. Four wells (green color) contained positive controls with 0.6 ng of DNA added as described in single-virus genomics protocol in Martinez-Hernandez et al (2017)^5^. Reaction was monitored at 45ºC in a CLARIOSTAR (BG) high resolution fluorimeter.


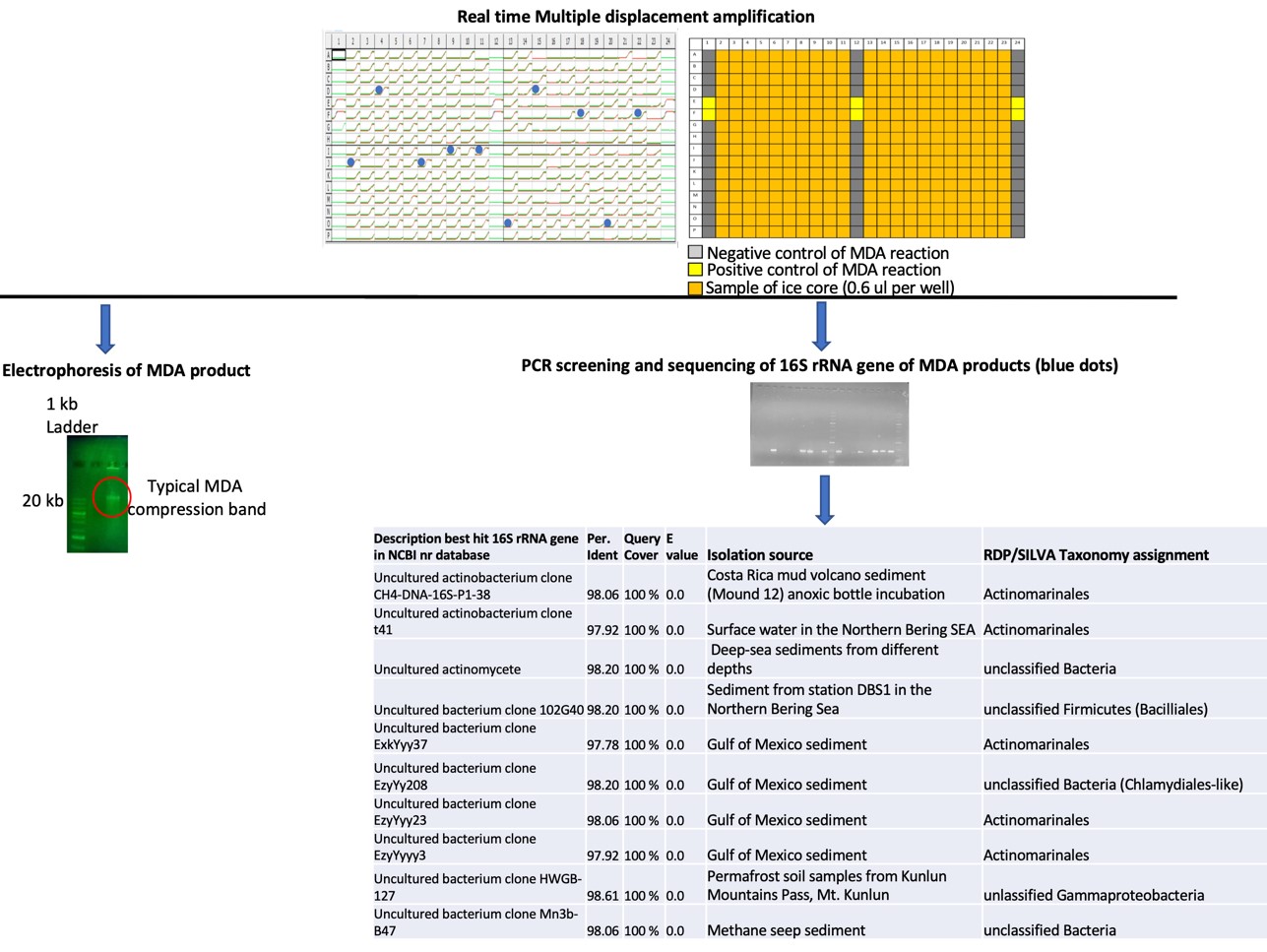


**Figure S7.** Data from real time multiple displacement amplification of the marine ice sample B15. MDA was performed in 384-multi-well plates. Layout with negative and positive controls and sample wells is depicted. All negative controls of MDA (columns 1, 12 and 24, except wells E1, F1, E24, and F24 that correspond to positive controls) showed no amplification indicating that reagents and procedures during ice core melting and whole genome amplification did not introduce external DNA contaminants. Negative controls contain all reagents except melted seawater (0. 6 ul) from the marine ice. Electrophoresis image in the left shows the common high molecular band observed typically from MDA products after amplification. Data from 16S rRNA gene PCR screening and sequencing of some randomly screened wells with successful MDA amplification (depicted with blue dots) are shown. The obtained 16S rRNA gene sequences were quality trimmed with Geneious and compared against nr database of Genbank and also with SILVA and RDP database.


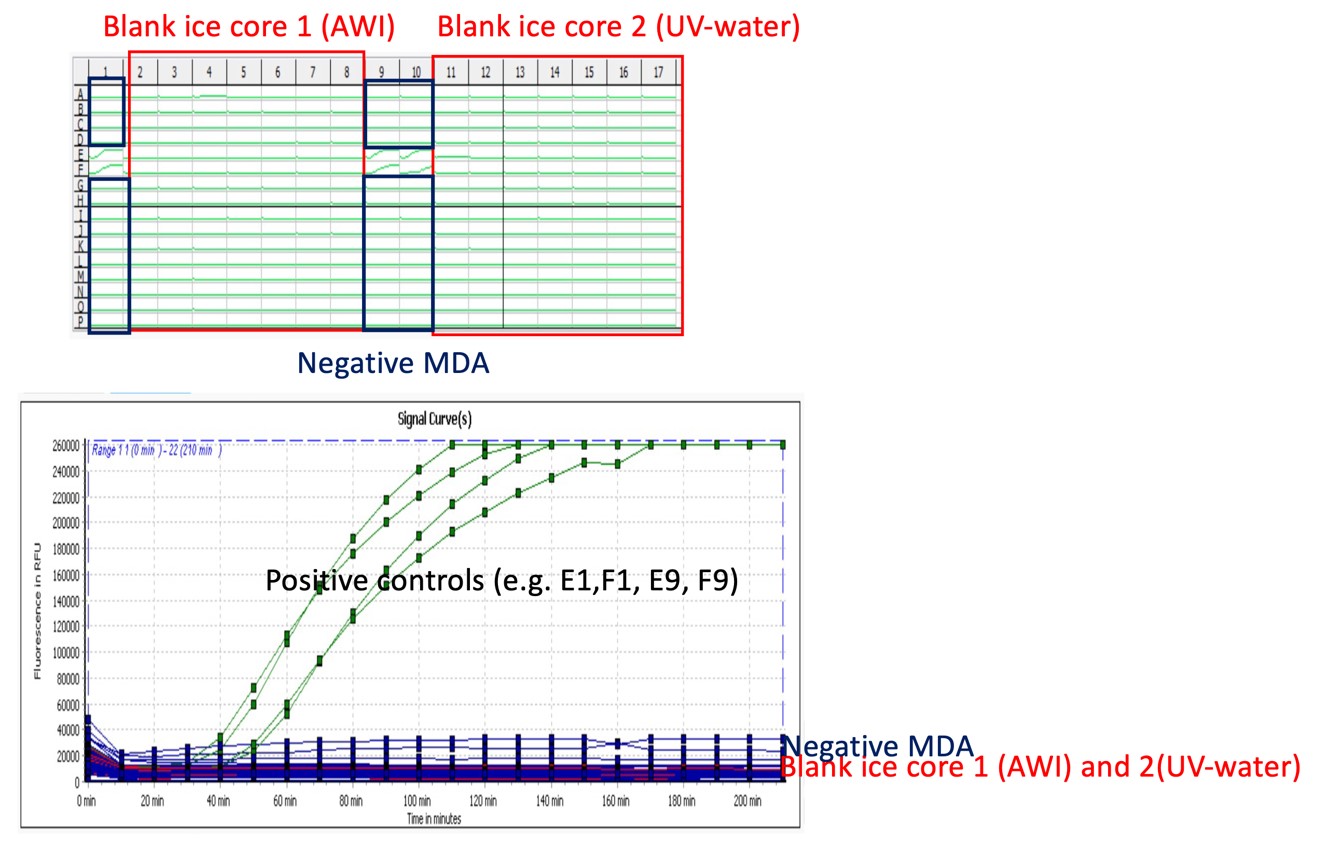


**Figure S8.** Real time multiple displacement amplification of blank ice core 1 (AWI) and 2 (UVwater). Blank ice core 1 (AWI) was made of sterile mQ water at the Alfred Wegener Institute and processed identically as the rest of samples. Blank ice core 2 (UV-water) was made of autoclaved mQ water UV for 15 min in a Stratalinker at the University of Alicante. Both blank ice cores were useful to monitor contamination during the whole ice core processing at AWI (cutting of ice core B15) and University of Alicante (e.g. ice core decontamination and MDA process) that could reach the inner part of the ice core. Wells containing melted inner part of blank ice cores are indicated in columns 2-8 for blank ice core 1 AWI and columns 11-17 for blank ice core 2 UA. Negative controls of MDA are displayed in blue, and positive controls containing 0.6 ng of DNA correspond to wells E1, F1, E9, F9, E10 and F10 as in Martinez-Hernandez et al (2017)^5^. Reaction was monitored at 45ºC in a CLARIOSTAR (BG) high resolution fluorimeter. None of the wells contained melted water from inner ice blank core 1 and 2 resulted in positive amplification indicating that foreign DNA potentially present or added by manipulation reached the inner part of the ice core B15 sample.

**Suppl. Table 1**: Data from Illumina sequencing, Trimming, Nonpareil, Mash and SpadesSC Quast and Binners. PlteCore_1(1), PlteCore_1(2), PlteCore_1(3) are tree library replicates from the same sampling point. 1/3 PlteCore_1 is another replicate from the same sampling point but just 1/3 plate wells sequenced. PlteCore_2 is other sampling point different than PlteCore_1 and 1/3 of the well plate. PlteCore_3 is the same sampling point as PlateCore_2 but 30x concentrate and 1/3 of the well plate.

| Sample name | Gb  (Raw data) | No. of reads | Trimmed Reads | | Diversity  Nonpareil | Estimate coverage Mash (%) | Assembled contigs SPAdes SC Quast | N50  SPAdesSC Quast | | Binned Contigs | | Number of bins  DAS tools | | |
| --- | --- | --- | --- | --- | --- | --- | --- | --- | --- | --- | --- | --- | --- | --- |
| PltCore_1 (1) | 5,8 | 38,311,280 | | 94,9% | 14.29 | 65.5 | 13978 | 13091 | 294 | | | | 4 | |
| PltCore_1 (2) | 1,5 | 9,622,094 | | 96,8% | 13.8 | 30.7 | 10682 | 7925 | 196 | | | | 2 | |
| PltCore_1 (3) | 6,9 | 45,519,646 | | 96,5% | 14.38 | 68.8 | 14177 | 10017 | 1505 | | | | 7 | |
| PltCore_1 | 6,6 | 43,774,964 | | 90,7% | 13.5 | 98.6 | 7377 | 12074 | 276 | | | | 3 | |
| PltCore_2 | 7 | 46,216,340 | | 92,5% | 14.58 | 80.3 | 11710 | 14974 | 362 | | | | 2 | |
| PltCore_3 | 6,8 | 45,179,010 | | 90,7% | 13.92 | 80.2 | 7933 | 14936 | | | 486 | | | 4 |

**Suppl. Table 2**: Data of bins obtained with Das Tool and classified with GTDB-Tk.

| Sample name | Sample genomes | Phylum | Lowest taxonomy | MAGs with same lowest taxon in Ross ^6^ Ice Shelf study | Completeness | Contamination (CheckM) |
| --- | --- | --- | --- | --- | --- | --- |
| PltCore_1 | PltC1_1.2 | Dependentiae | Vermiphilaceae | - | 72.55 | 3.3 |
|  | PltC1_3.3 | Dependentiae | Vermiphilaceae | - | 90.2 | 3.86 |
|  | 1_3_PltC1.1 | Dependentiae | Vermiphilaceae | - | 64.71 | 0.0 |
|  | PltC1_1.1 | Desulfobacterota | Dadabacteria | - | 84.31 | 0.84 |
|  | PltC1_3.1 | Desulfobacterota | Dadabacteria | - | 74.51 | 1.72 |
|  | PltC1_3.5 | Actinobacteriota | Acidimicrobiia | - | 66.67 | 10 |
|  | PltC1_1.3 | Actinobacteriota | Spongiisociudales | RIS_MAG_141 | 86.27 | 7.26 |
|  | PltC1_2.1 | Actinobacteriota | Spongiisociudales | RIS_MAG_141 | 70.59 | 4.27 |
| PltCore_2 | PltC3.4 | Chlamydiota | Rhabdochlamydiaceae | - | 86.27 | 4.39 |
|  | PltC2.2 | Actinobacteriota | Spongiisociudales | - | 76.47 | 0.43 |
|  | PltC3.3 | Proteobacteria | Xanthomonadales | - | 82.35 | 8.1 |

12

# References

1. Mouginot, J., Rignot, E. & Scheuchl, B. Continent-Wide, Interferometric SAR Phase, Mapping of Antarctic Ice Velocity. *Geophys. Res. Lett.* **46**, 9710–9718 (2019).
2. MEaSUREs Phase-Based Antarctica Ice Velocity Map, Version 1 | National Snow and Ice Data Center. https://nsidc.org/data/nsidc-0754/versions/1.
3. Adusumilli, S., Fricker, H. A., Medley, B., Padman, L. & Siegfried, M. R. Interannual variations in meltwater input to the Southern Ocean from Antarctic ice shelves. *Nat. Geosci.* **13**, 616–620 (2020).
4. Alley, K. E. *et al.* Continent-wide estimates of Antarctic strain rates from Landsat 8-derived velocity grids. *J. Glaciol.* **64**, 321–332 (2018).
5. Martinez-Hernandez, F. *et al.* Single-virus genomics reveals hidden cosmopolitan and abundant viruses. *Nat. Commun.* **8**, 15892 (2017).
6. Martínez-Pérez, C. *et al.* Phylogenetically and functionally diverse microorganisms reside under the Ross Ice Shelf. *Nat. Commun. 2022 131* **13**, 1–15 (2022).
